# Early-Life Antibiotics and Childhood Obesity: Yeast Probiotics as a Strategy to Modulate Gut Microbiota

**DOI:** 10.7759/cureus.36795

**Published:** 2023-03-28

**Authors:** Sanjiv Singh Rawat, Nigam P Narain, Sanjay M Marathe, Sourabh B Sonawale, Krishna C Veligandla

**Affiliations:** 1 Pediatrics, Child Care Hospital, Indore, IND; 2 Pediatrics, Patna Medical College, Patna, IND; 3 Pediatrics, Colours Pediatrics Intensive Care and Neonatal Care Hospital, Nagpur, IND; 4 Medical Affairs, Dr. Reddy’s Laboratories, Hyderabad, IND

**Keywords:** overweight, obesity, gut microbiome, children, antibiotics

## Abstract

This study aimed to review the existing literature to investigate the potential link between early-life antibiotic use and being overweight or obese in children. PubMed, Web of Science, Embase, Google Scholar, and Cochrane Library were searched to identify studies published until August 2021 that assessed the relationship between early-childhood antibiotic use and measures of body mass index. The studies included children aged 0-18 years. Only cohort studies were taken into consideration. Studies published in languages other than English were excluded. Antibiotic usage in early life may increase the risk of obesity in children and the addition of yeast probiotics, such as *Saccharomyces boulardii CNCM I 745*, to antibiotic prescription can serve as a potential option to mitigate this risk.

## Introduction and background

Obesity is a complicated disease involving the interplay between biological, developmental, environmental, behavioral, and genetic variables, leading to an energy imbalance and the accumulation of extra adipose tissue [1,2]. The worldwide prevalence of obesity more than doubled between 1990 and 2015, and its rates have risen dramatically in recent years owing to a global shift toward an obesogenic environment [3-5].

In many countries, obesity in children is rising faster than in adults [3]. Approximately 10% of school-going children worldwide aged 5-17 are either obese or overweight, with a prevalence ranging from 30% in the United States to less than 2% in Sub-Saharan Africa. According to 2016 estimates, obesity has grown 10-fold, from 11 million to 124 million, among school-going children and adolescents in only three decades [4,5]. Childhood obesity can elevate the risk of morbidity later in life, regardless of whether it continues into adulthood. Hypertension, type 2 diabetes, dyslipidemia, left ventricular hypertrophy, non-alcoholic steatohepatitis, obstructive sleep apnea, and orthopedic and psychological issues are all associated with pediatric obesity [1,5].

In India, stunting and underweight exist alongside overweight and obesity in children, creating a dietary conundrum. The prevalence of stunting, wasting, and being underweight in children aged five years was found to be 38%, 21%, and 36%, respectively, in the National Family Health Survey-4 (2015-2016). According to the study, 2% of Indian children under the age of five years were overweight [5]. The prevalence of overweight/obesity among adolescent Indian children increased from 9.8% to 11.7% between 2006 and 2009 [6]. Lobstein and Jackson-Leach predicted India will have 17 million obese children by 2025; this tendency has been documented in both urban and rural regions [5].

In addition to genetic and environmental variables, gut microbiota plays an essential role in the development of obesity. Gut microbiota dysbiosis, an imbalanced or disordered gut microbial ecology, impacts obesity etiology by affecting energy harvest, nutrition metabolism, inflammatory pathways, and the gut-brain axis [7-10].

Antibiotics significantly contribute to altering the gut microbiota. They are regularly administered to infants and children, and up to 40% of infants are exposed to them either directly or indirectly through maternal intrapartum antibiotic prophylaxis [11]. In the United States, usually, children complete three courses of antibiotics by the age of 2 and 10 courses by the age of 10, with many of these courses prescribed for viral illnesses and, therefore, providing no therapeutic benefit [11,12]. From 2000 to 2015, India’s antibiotic consumption increased by 103%, making it the highest among low- and middle-income economies [13]. Antibiotics decrease the total diversity of the intestinal microbiota, including the loss of certain key taxa, resulting in metabolic changes, increased vulnerability of the gut to colonization, and the development of bacterial antibiotic resistance [14]. Even short-term or low-dose antibiotic therapy can permanently alter the gut microbiota and affect long-term health consequences [15].

Objective

The key objective of this review was to summarize the research investigating the potential link between antibiotic use and obesity/overweight in children. In addition, we aimed to review the role of probiotics in managing dysbiosis to avoid future adverse effects such as obesity.

## Review

**Figure 1:**
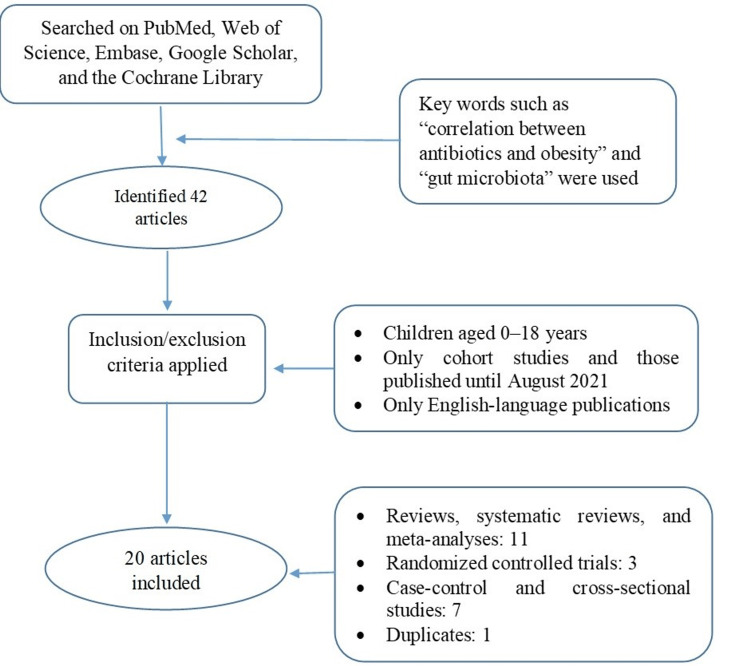
Consort flow diagram.

For this review, a data search was conducted on PubMed, Web of Science, Embase, Google Scholar, and the Cochrane Library to identify studies published until August 2021 that assess the relationship between early-childhood antibiotic use and the body mass of the child. Search terms such as “antibiotics,” “gut microbiota,” “overweight,” and “obesity” were used. The inclusion criteria included children aged between 0 and 18 years, and only cohort studies and English-language publications were considered. Studies on antibiotics of any kind, used for any duration, any method, or for any reason were recorded. Studies based on children who were extremely malnourished, affected by an underlying disease, born with low birth weight, or who were small for their gestational age were excluded (Figure 1).

The link between dysbiosis and obesity

The following hypotheses have been proposed to explain how antibiotics contribute to obesity/overweight [16,17]: (1) Gut bacteria’s capacity to absorb energy from indigestible polysaccharides has improved. (2) The gut microbiota that defends against obesity from a metabolic standpoint has reduced. (3) Intestinal defenses and immunity have weakened. (4) Hepatic lipogenesis is altered. (5) Metabolic signaling has changed.

Antibiotic use early in life has been linked to a decrease in the quantity of beneficial gut flora, leading to reduced gut microbial diversity and lower rates of colonization of protective commensal bacteria. An increase in Enterobacteriaceae (phylum Proteobacteria) and a decrease in good bacteria, such as Bifidobacteria and butyrate markers, have been documented. Short-chain fatty acids (SCFA), which have beneficial effects on mammalian energy metabolism, are produced by Bifidobacteria (phylum Actinobacteria) and Lactobacilli (phylum Firmicutes) [16,17].

Antibiotic use has been linked to an increase in Bacteroidetes and Proteobacteria, with some species in these phyla associated with severe infections. *Bacteroides *spp. may offer some protection against invasive pathogens; however, *Bacteroides *have also been linked to bloodstream infections and the development of abscesses [17,18].

**Figure 2:**
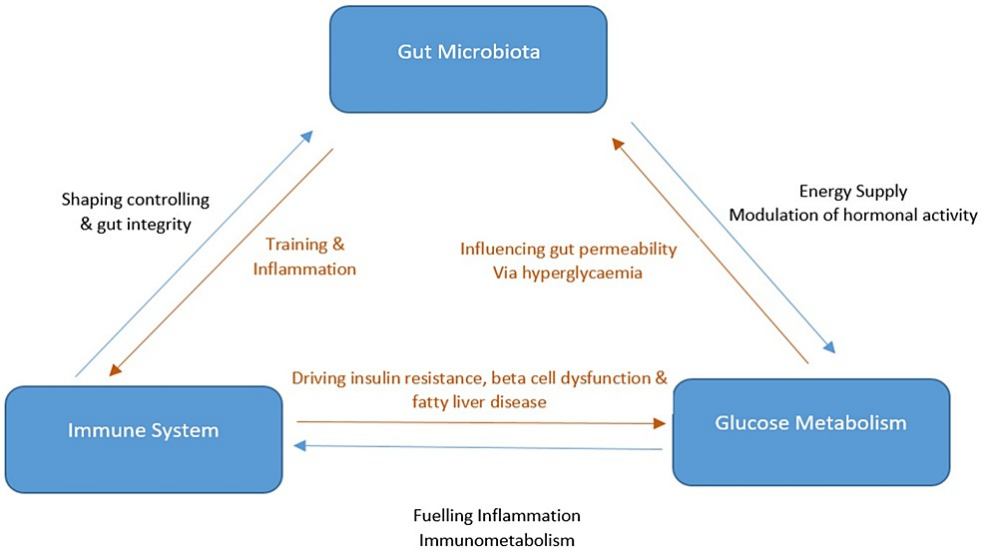
Three-way interactions among the gut microbiota, glucose metabolism, and immune system.

Bifidobacteria protects against obesity and its metabolic consequences, partially by improving gut barrier function, which decreases metabolic endotoxemia, the abundance of bacterially generated lipopolysaccharides (LPS) in the blood. Once the ratio of Bifidobacteria to Bacteroides gets perturbed, the abundance of barrier function-improving microbes drops and the number of LPS-producing organisms increases [19,20]. LPS in the bloodstream causes inflammation, insulin resistance, and weight gain, and is thought to play a role in the development of obesity and associated diseases, as shown in Figure 2 [21]. Bifidobacteria can enhance metabolic health by reducing LPS leakage from the gut, probably by upregulating tight junction proteins [19-21].

The synthesis of SCFA via colonic fermentation, which involves the anaerobic degradation of food fiber, protein, and peptides, helps the gut bacteria contribute to energy metabolism. SCFAs are bacterial waste products generated to maintain a healthy redox balance in the gut. Acetate, propionate, and butyrate are the most common SCFA species. The Bacteroidetes phylum produces acetate and propionate, whereas the Firmicutes phylum produces butyrate. Body weight, glucose homeostasis, and insulin sensitivity have all been found to benefit from them [17,19-21].

Is There Any Evidence That Antibiotics Have the Potential to Cause Obesity or Weight Gain?

We found 20 observational studies encompassing a total of 1,412,275 participants that allowed us to assess the association between antibiotic exposure before the age of four years and later measurements of body mass or the risk of obesity/overweight in childhood (Table 1). Both prospective and retrospective cohort studies were included in the review. Most studies were conducted in the United States and Europe. The overall exposure window in the studies was 0-48 months of age.

**Table 1:** Characteristics of 20 cohort studies on the association between antibiotic exposure in early life and the risk of childhood overweight and obesity.

Study	Year of publication and location	Study design	Number of subjects	Exposure time	Outcome
Ajslev et al. [22]	2011 Denmark	Prospective cohort	28,354 children from the Danish National birth cohort	Infancy (<6 months)	Antibiotic use during the first 6 months of life led to an increased risk of being overweight among children of normal-weight mothers (odds ratio (OR) = 1.54, 95% confidence interval (CI) = 1.09–2.17)
Murphy et al. [23]	2013 New Zealand	Prospective cohort	871 European children	Infancy (<12 months)	Antibiotic exposure at <1 year was not associated with any significant differences in the percentage of body fat or body mass index (BMI) z-score at 3, 7, or 11 years in those born small for their gestational age (SGA), non-SGA, or combined groups
Azad et al. [24]	2014 Canada	Prospective cohort	616 children	Infancy (<12 months)	Infants receiving antibiotics in the first year of their lives were more likely to be overweight later in childhood compared with those who were unexposed (32.4% vs. 18.2% at age 12, p = 0.002)
Trasande et al. [25]	2013 United Kingdom	Prospective cohort	11,532 children	Infancy (<6 months)	Antibiotic exposure during the earliest time window (<6 months) was consistently associated with increased body mass (+0.105 and +0.083 standard deviation (SD) unit increase in weight-for-length z-scores at 10 and 20 months, p < 0.001 and p = 0.001, respectively)
Bailey et al. [26]	2014 United States	Prospective cohort	64,580 children	Infancy (<24 months)	Cumulative exposure to antibiotics was associated with later obesity (rate ratio (RR) = 1.11; 95% CI = 1.02–1.21 for four episodes); this effect was stronger for broad-spectrum antibiotics (RR = 1.16; 95% CI = 1.06–1.29)
Dawson-Hahn and Lampe [27]	2015 Washington, United States	Longitudinal birth cohort	4,938 children	0–47 months	Antibiotic exposure was associated with overweight development (OR = 1.03, 95% CI = 1.01–1.05). Children had 3% higher odds of overweight development at 48–59 months for each additional course of antibiotics during 0–47 months
Saari et al. [28]	2015 Finland	Prospective cohort	12,062 children	Infancy (<24 months)	Exposed children were on average heavier than unexposed children (adjusted BMI for age z-score difference in boys was 0.13 SD (95% CI = 0.07–0.19, p = 0.001) and in girls was 0.07 SD (95% CI: 0.01–0.13, p = 0.05)
Scott et al. [29]	2016 United Kingdom	Retrospective cohort	21,714 children	Infancy (<24 months)	Antibiotic exposure was associated with an increased risk of obesity at 4 years (OR = 1.21, 95% CI = 1.07–1.38). ORs increased with repeated exposure
Mbakwa et al. [30]	2016 Netherlands	Prospective cohort	979 children	Infancy (<24 months)	Associations with weight z-scores were mainly driven by exposure to broad- (≥2 courses, adj β = 0.11; 95% CI = 0.003–0.22) and narrow-spectrum β-lactams (1 course, adj β = 0.18; 95% CI = 0.005–0.35) during the follow-up period
Schwartz et al. [31]	2016 Baltimore, United States	Longitudinal cohort	142,824 children	Year before each BMI	A persistent association, which got stronger with increasing age (p < 0.001), was observed. The addition of the progressive association among children with at least three BMIs (n = 79,752) revealed that higher cumulative orders were associated with progressive weight gain; this did not vary by age
Gerber et al. [32]	2016 United States	Retrospective cohort	38,522 children	Infancy (<6 months)	Antibiotic exposure was not significantly associated with the rate of weight change (0.7%; 95% CI = −0.1%–1.5%; p = 0.07, equivalent to approximately 0.05 kg; 95% CI = −0.004–0.11 kg of added weight gain between the ages of 2 years and 5 years)
Li et al. [33]	2017 United States	Retrospective cohort	260,556 children	Infancy (<12 months)	After controlling for maternal age, race or ethnic origin, pre-pregnancy BMI, preterm delivery, low birth weight, maternal antibiotic use, and infection during pregnancy, infection without antibiotic use in infancy was associated with an increased risk of childhood obesity compared with controls without infection (OR = 1·25, 95% CI = 1.20–1.29)
Ville et al. [34]	2017 United States	Prospective cohort	97 Latino children	Infancy (<6 months)	Early antibiotic exposure (before 6 months of age) was independently associated with an increased risk for rapid infant weight gain (OR = 6.42, 95% CI = 1.17–35.06) and obesity at age 2 (OR = 6.15, 95% CI = 1.03–36.70)
Rogawski et al. [35]	2017 Multi-country	Birth cohort study	1,954 children	0–6 months	Compared with unexposed children, two or more courses of metronidazole, macrolides, and cephalosporins were associated with adjusted increases in weight for age of 0.24 (95% CI = 0.04–0.43), 0.23 (95% CI = 0.05–0.42), and 0.19 (95% CI = 0.04–0.35) between 6 months to 2 years, respectively
Block et al. [36]	2018 Boston	Multi-institutional national cohort	362,550 children	<24 months	For children without a complex chronic condition at 5 years, we estimated a higher mean BMI z-score by 0.04 (95% CI = 0.03–0.05) and higher odds of being overweight or obese (OR = 1.05; 95% CI = 1.03–1.07) associated with obtaining any (versus no) antibiotics
Stark et al. [37]	2018 United States	Retrospective cohort	241,502 children	0–24 Months	Antibiotic prescriptions were associated with obesity (hazard ratio = 1.26; 95% CI = 1.23–1.28). This association persisted regardless of antibiotic class and strengthened with each additional class of antibiotic prescribed
Kelly et al. [38]	2018 Ireland	Irish National Longitudinal Study of Children	8,186 children	Infancy	Any antibiotic usage between 2 and 3 years did not predict the risk of being overweight or obese at age 5. Four or more courses of antibiotics between 2 and 3 years were independently associated with obesity at age 5 (OR = 1.6, 95% CI = 1.11–2.31)
Aversa et al. [39]	2020 Olmsted county	Retrospective cohort	14,572 children	Infancy (<24 months)	Early antibiotic exposure was associated with an increased risk of childhood-onset asthma, allergic rhinitis, atopic dermatitis, celiac disease, overweight, obesity, and attention-deficit hyperactivity disorder (hazard ratios of 1.20–2.89; p < 0.05 for all)
Uzan-Yulzari et al. [40]	2021 Finland	Birth cohort	12,422 children	1 month	The cumulative number of antibiotic courses after the neonatal period but during the first 6 years of life was associated with significantly higher BMI z-scores throughout the study period in both boys and girls (p < 0.001) in a hierarchical linear mixed model for repeated measurements
Aris et al. [41]	2021 United States	Retrospective cohort	183,444 children	0–48 months	Exposure to any antibiotics at 0–47 months of age (vs. no exposure) was associated with an earlier age (−0.60 months (95% CI = −0.81–−0.39 months) and higher BMI at rebound (β coefficient = 0.02 (95% CI = 0.01–0.03). These associations were the strongest for children with at least four episodes of antibiotic exposure

Of the 20 studies, 17 had overall measures of association. Among the remaining three studies that involved a total of 299,949 participants, one study had the most participants (260,556 children) and showed that association with infection, but not with antibiotic usage, was linked to an increased risk of childhood obesity.

The 17 studies comprising a total of 1,112,326 participants clearly highlighted that the use of antibiotics was linked to obesity. Antibiotic exposure in early infancy was related to a higher body mass index (BMI) and a higher prevalence of obesity in healthy children, according to these cohort studies.

Aversa et al. demonstrated that antibiotic exposure during childhood was associated with an increased risk of childhood obesity, asthma, allergic rhinitis, atopic dermatitis, celiac disease, and attention-deficit hyperactivity disorder. The hazard ratios were 1.20-2.89, with a p-value <0.05 for all conditions. This study was conducted and published by the Mayo Clinic [39].

Uzan-Yulzari et al. showed that antibiotic exposure during the neonatal phase was linked to a long-term disruption of the gut microbiota, which may cause stunted development in boys during the first six years of their life, whereas antibiotic usage later in childhood was linked to an increase in BMI. In most studies, antibiotic exposure occurred during the first two years of life and was significantly associated with weight gain/obesity [40].

Another recent study by Aris et al. on 0-48-month-old children documented that antibiotic exposure was associated with statistically significant BMI trajectory milestones during infancy and early childhood. These associations were the strongest for children with at least four episodes of antibiotic exposure [41].

Is There Any Treatment Option Available to Prevent the Consequences Associated With Dysbiosis?

Probiotics seem to be a promising option to treat dysbiosis and are well-tolerated upon long-term usage. The World Health Organization defines probiotics as “live microorganisms which when administered in adequate amounts confer a health benefit on the host.” Probiotics are continually used to preserve human intestinal health by improving the equilibrium of internal microbiota. As a result, the number of harmful bacteria that cannot live in acidic environments decreases, whereas helpful bacteria that thrive in such environments multiply, thereby balancing the gut microbiota [42].

Probiotics are available in various forms, the most common being bacterial and yeast probiotics. Only a few of the many available probiotics have scientific merit and global recommendations. *Lactobacillus rhamnosus GG* is a bacterial probiotic, whereas *Saccharomyces boulardii CNCM I 745* is a yeast probiotic. Both have been extensively studied and are recommended as adjunct therapy while managing acute gastroenteritis and antibiotic-associated diarrhea (AAD) in children [43,44].

AAD is characterized by the passage of loose, watery stools three or more times a day following the intake of antibiotics used to treat bacterial illnesses. It affects 5%-30% of patients, either early in the course of antibiotic medication or up to two months after the treatment is completed [44,45]. Although AAD is not evident in every patient, antibiotic intake is still a cause of gut dysbiosis. AAD is the first visual symptom of dysbiosis and both *S. boulardii CNCM I 745* and *L. rhamnosus GG* can be used along with antibiotics for preventing it [46,47].

Probiotics should be administered along with antibiotics and should be continued for a few days after stopping antibiotics to obtain the desired results. However, the concurrent administration of *L. rhamnosus GG* and antibiotics faces the challenge that the antibiotic may potentially kill the bacterial probiotics. Hence, the two must be administered at least two to four hours apart. On the contrary, a time gap is not required while using *S. boulardii CNCM I 745*. Further, yeast probiotics are 10 times larger in size than bacteria and can adhere firmly to the epithelial layer of the intestine, precluding the adhesion of any pathogenic bacteria [44,46,47]. *S. boulardii CNCM I 745* neither shows resistance to antibiotics nor plays any role in genetic transfer [48].

*S. boulardii CNCM I 745* has several established mechanisms of action [46,47]. It shows antitoxic effects against *Clostridium difficile* toxins A and B, cholera toxin, and *Escherichia coli*. It can interfere with gut infections either directly or indirectly. For example, it can directly suppress the growth of *Salmonella typhimurium*, *Yersinia enterocolitica*, and *Aeromonas hemolysin*. It can enhance the production of SCFAs, which are depleted during illness, suggesting altered intestinal fermentation. It can decrease mucositis, repair fluid transport routes, promote protein and energy synthesis, or serve as a trophic agent by producing spermine and spermidine, as well as other brush border enzymes that help enterocytes mature. Finally, it can modulate immune responses by acting as a stimulant or by decreasing pro-inflammatory responses. Intestinal secretory immunoglobulin A (IgA) levels may rise because of *S. boulardii* infection. It is also linked to elevated levels of blood IgG against *C. difficile* toxins A and B. It may disrupt nuclear factor-κB-mediated signaling pathways, which promote the generation of pro-inflammatory cytokines.

Some clinical studies that used *S. boulardii CNCM I 745* along with antibiotics have shown that it helps prevent and manage dysbiosis. Additional details are presented in Table 2.

**Table 2:** Details of studies performed on Saccharomyces boulardii CNCM I 745 along with antibiotics.

Study	Year of publication and location	Study title	Study design	Conclusion
Selig et al. [48]	2020, United States	*Saccharomyces boulardii CNCM I-745* probiotic does not alter the pharmacokinetics of amoxicillin	A traditional pharmacokinetic study in mice	Pharmacokinetic parameters, such as half-life, the area under the curve, peak concentrations, time to reach maximum concentration, and the elimination rate constant of amoxicillin, did not significantly differ between the probiotic-treated and untreated control groups
Kabbani et al. [49]	2016, United States	A prospective randomized controlled study on the effects of *Saccharomyces boulardii CNCM I-745* and amoxicillin-clavulanate or the combination on the gut microbiota of healthy volunteers	Prospective randomized controlled trial, 53 healthy subjects enrolled in the study	*Saccharomyces boulardii CNCM I-745* was effective in preventing or reducing antibiotic-associated changes in colonic microbiota when given concomitant with or subsequent to antibiotic therapy
Monjaraz et al. [50]	2021, Mexico	Gut microbiota in Mexican children with acute diarrhea: an observational study	A single-center observational study including 30 children (6 months to 5 years old)	Acute diarrhea was accompanied by significant alterations in the gut microbiota. *Saccharomyces boulardii CNCM I-745* treatment may facilitate gut microbiota restoration in children with acute diarrhea, mostly by improving the alpha diversity

A study of 60 women treated for bacterial vaginosis analyzed the effects of antibiotic therapy alone, concurrently with S. boulardii treatment, or followed by S. boulardii treatment. In the study, 60 women were given metronidazole (3 × 400 mg/day) and ciprofloxacin (2 × 500 mg/day) to treat bacterial vaginosis. Group I received antibiotics for the first and second weeks. Group II received 250 mg S. boulardii tid in addition to antibiotics for weeks one and two. Group III received antibiotics for weeks one and two, followed by 250 mg S. boulardii tid for weeks three and four. Fluorescence in situ hybridization analysis of Carnoy fixated stool cylinders was done from days -90, -60, -30, 7, 14, 28, 42, 56, and 70.

**Figure 3:**
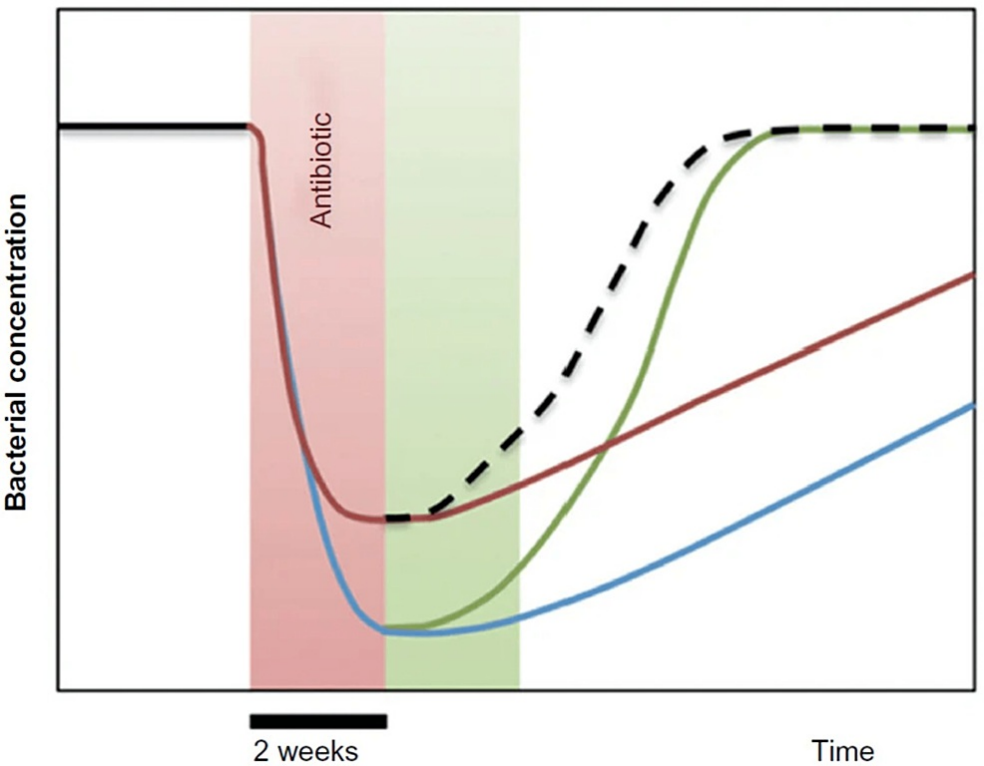
Generalized scheme of the effects of antibiotic dysbiosis on habitual/essential bacteria and other substantial bacteria.

As shown in Figure 3, the main microbial population is drastically reduced (blue line) after two weeks of antibiotic therapy (red area). *S. boulardii* (red area; red line) provided during antibiotic therapy can mitigate this loss by preserving the microbiome. If *S. boulardii* is given after antibiotic therapy (green area; green line), it can help the microbial community regenerate more quickly. As a result, a combination of both, with* S. boulardii* therapy during and after antibiotic treatment, would be ideal. The hypothetical black dotted line drawn from the other lines represents this. The worst-case situation is that no *S. boulardii* therapy is provided (blue line) (Figure 3).

Although stable enterotypes were seen before treatment, the antibiotics led to the suppression of the habitual/essential (most prevalent) bacteria as well as other significant bacterial groups. If given concurrently with an antibiotic, *S. boulardii* might reduce the suppression of these bacteria caused by the antibiotic. After receiving antibiotic therapy, *S. boulardii* dramatically accelerated the return of the natural microbiota. *S. boulardii* also decreased the number of pre- and post-mismatches in the microbial community. Contrary to what would have happened if *S. boulardii* had been provided, antibiotic therapy greatly increased population differences (compared to before treatment) [46].

As this is an attempt to present a narrative review of the evidence, there are a few limitations to this review. We only considered cohort studies. Moreover, the age groups and study durations were different. Another important limitation is that we did not focus on any specific antibiotic while including studies in this review. Dosage regimens were different in all cohort studies. Finally, per the evidence, yeast probiotics are superior to bacterial probiotics, but head-to-head studies are very limited. Therefore, for establishing a strong correlation between antibiotics and obesity, we need further larger controlled studies.​​

## Conclusions

Antibiotics are very commonly used in the first few years of a child’s life, and there are many hypotheses linking early antibiotic exposure to childhood obesity. Antibiotics mainly impact the gut microbiota, and this altered gut microbiota increases BMI or obesity later in life. In this review, 17 studies showed an association between antibiotic usage in early life and obesity in later stages of life. Evidence-based yeast probiotics, *S. boulardii CNCM I 745* can be adjuvants to antibiotic therapy to modulate the gut microbiota.
